# Strength and Performance Enhancement of Multilayers by Spatial Tailoring of Adherend Compliance and Morphology via Multimaterial Jetting Additive Manufacturing

**DOI:** 10.1038/s41598-018-31819-2

**Published:** 2018-09-11

**Authors:** Jabir Ubaid, Brian L. Wardle, S. Kumar

**Affiliations:** 1 Department of Mechanical and Materials Engineering, Khalifa University of Science and Technology, Masdar Institute, Abu Dhabi, 54224 UAE; 2 Department of Aeronautics and Astronautics, Massachusetts Institute of Technology, Cambridge, MA 02139 USA

## Abstract

Material tailoring of bondlayer compliance is a known effective route to enhance performance of multilayers, and here spatial material-tailoring of compliance and morphology of the adherends is examined. Multimaterial jetting additive manufacturing (AM) allows us to realize for the first time compliance- and morphology-tailored adherends, and evaluate directly the mechanical performance, including failure, of the tensile-loaded multilayers. Adherend compliance-tailoring, unlike bondlayer tailoring, requires additional consideration due to adherend bending stiffness and moment influences on bondlayer stresses. We introduce anisotropic as well as layered/sandwich adherend tailoring to address this dependence. Numerical models show that for both sub-critical and critical bondlengths (at which shear-dominated load transfer occurs through the bondlayer), adherend tailoring reduces peak stresses significantly, particularly peel stress (reductions of 47–80%) that typically controls failure in such systems. At sub-critical bondlengths, the AM-enabled layered/sandwich adherend tailoring shows significantly increased experimental performance over the baseline multilayer: strength is increased by 20%, toughness by 48%, and strain-to-break by 18%, while retaining multilayer stiffness. The adherend tailoring demonstrated here adds to the techniques available to increase the performance of bonded multilayers, suggesting that adherend tailoring is particularly well-suited to additively manufactured multilayers, but can also have application in other areas such as layered electronics and advanced structural composite laminates.

## Introduction

Structural connections that are lightweight, cost-effective and compatible with a variety of dissimilar materials have led to the increased use, and interest in, multimaterial joining, with application to adhesive bonding in the fields of aerospace, automotive, microelectronics and biomedical systems. Multimaterial joining is particularly well-suited to additively manufactured parts that typically require joining due to sub-component level build volumes and dissimilar material combinations, or as a technique within AM build volumes to join dissimilar materials. The ability of emerging multimaterial additive manufacturing (AM) to tailor features of the joined structures to enable higher-performance joined multilayers in a single manufacturing process opens new opportunities to expand function and increase performance. Examples of tailoring enabled by multimaterial AM include modification of surface texture at the bonded interfaces^1^, geometrically interlocked layers^2–5^, and compliance tailoring of the bondlayer^6–9^.

**Figure 1:**
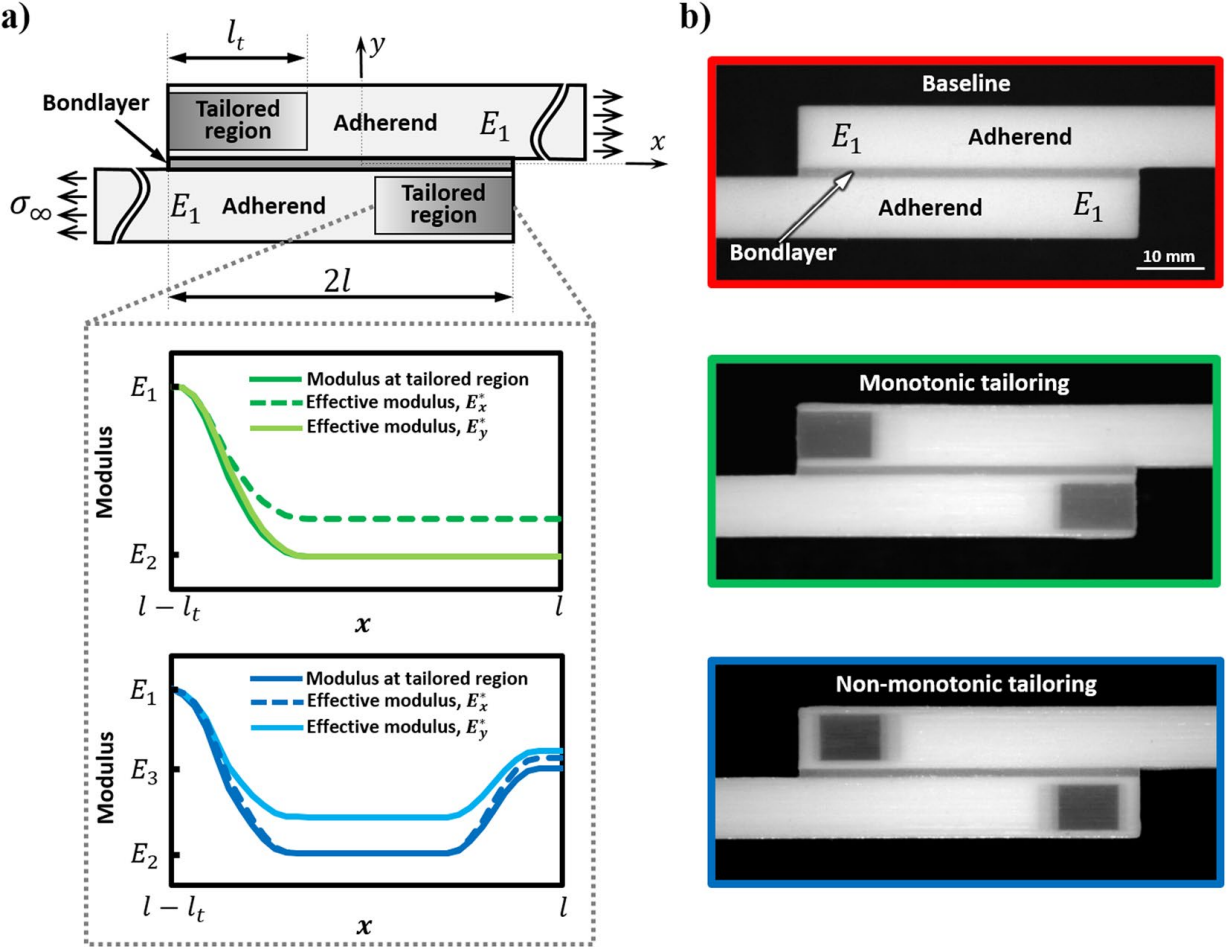
Additively manufactured multilayers with compliance- and morphology-tailored adherends in a single-lap joint configuration: (**a**) Conceptual representation of multilayer subjected to far field tensile stress σ_*∞*_ with two different choices of compliance- and morphology-tailoring over a length *l*_*t*_ from the overlap ends where *l*_*t*_/2*l* = 0.35. The plots show modulus variation in the tailored region as well as the effective directional moduli variation in the adherend (E^*^_x_(*x*) and E^*^_y_(*x*)) for monotonic tailoring and non-monotonic tailoring, where Young’s modulus in the tailored region varies between *E*_1_ (the baseline adherend modulus) and *E*_2_(*E*_1_ ≅ 3000*E_2_). (**b**) Side view optical images of the AM multilayers.

**Figure 2:**
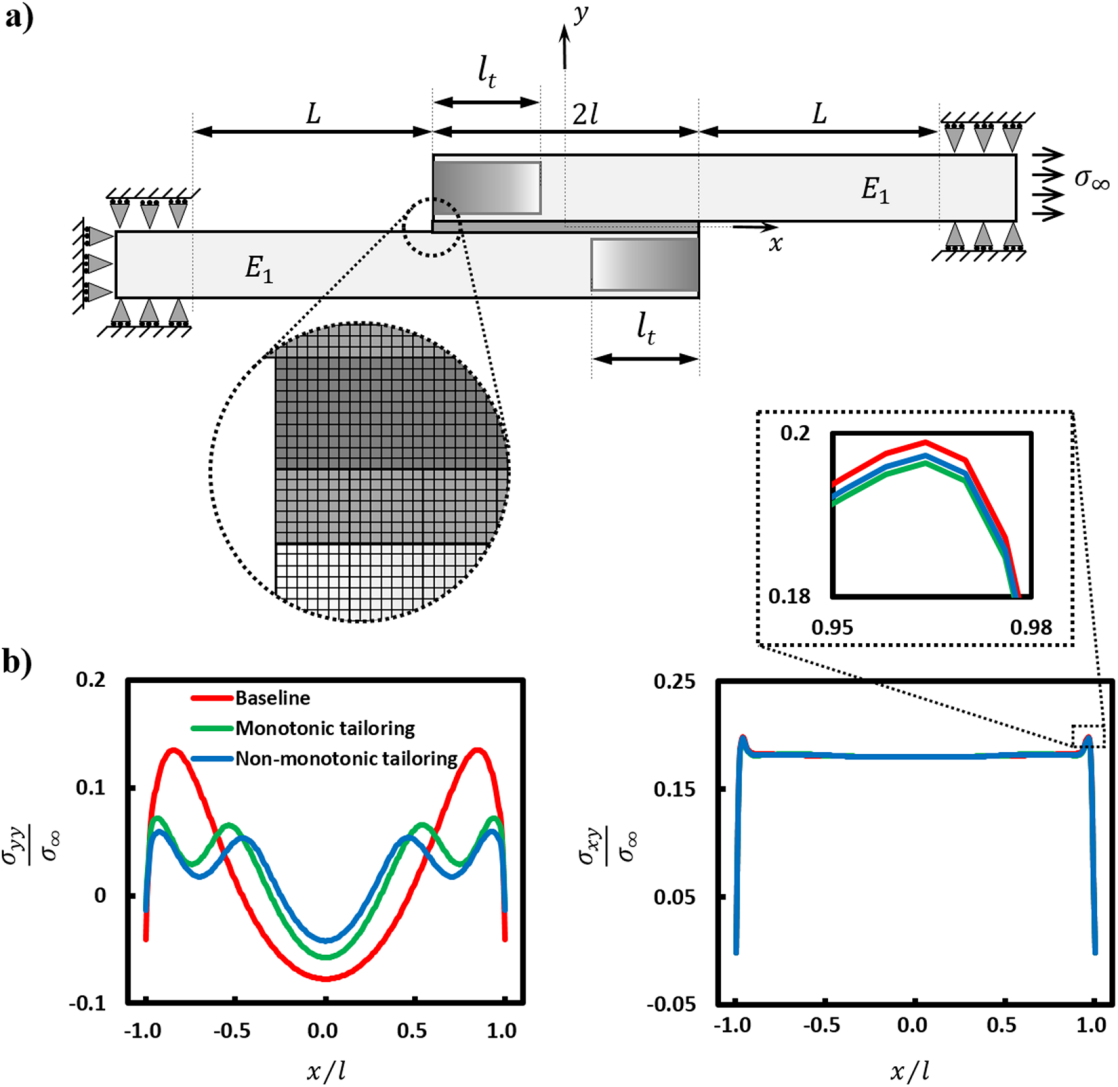
Numerical models and stress results in the bondlayer of the multilyaer SLJ: (**a**) Finite element model of the SLJ multilayer under far field stress ${\sigma }_{\infty }$ with boundary conditions that replicate mechanical testing, together with an inset of the refined mesh in the sress concentration region near the free edge of the adherends. (**b**) Peel and shear stress distribtution in the midline of the bondlayer over the bondlength for baseline and tailored (monotonic and non-monotonic) configurations under a tensile load of 200 N (see Fig. 3).

The aforementioned AM-based approaches to improving multi-material multilayer performance all seek to reduce the stress concentrations in the bondlayer near the free edges of the adherends (joined layers) in multilayer systems that include single- and double-lap joint configurations. These stress concentrations drive failure in the multilayers and arise due to material mismatch and geometric discontinuities. Single lap joints (SLJs) are an important multilayer configuration and the most commonly encountered bonded system configuration. Stresses in the bondlayer of the SLJ (see Fig. 1a), including transverse stress (σ_*yy*_), oftentimes termed ‘peel stress,’ and shear stress (σ_*xy*_), both concentrate near the free edges of the adherends (see Fig. 2b for exemplary stress profiles)^6,10^. These multilayers are usually designed to have critical bondlengths so as to enable load-transfer predominantly through shearing of the bondlayer^11–13^, although practical design constraints oftentimes require SLJs with sub-critical bondlengths, as considered herein. Although adherend failure is possible, particularly in some advanced composites, typically failure (adhesive and/or cohesive) is associated with the less-structural bondlayer due to these stress concentrations^14–16^. Increased performance is realized by either reducing the stress concentrations, or improving the strength and toughness (generally mutually exclusive properties^17^) of the bondlayer. Techniques to reduce stress concentrations, some purely theoretical and some impractical, include alteration of the bondlayer or its geometry (e.g., spew fillet), employing a compliance-tailored bondlayer, interlocking adherends, etc.^6,10,18–27^. Several studies on modifying the adherend geometry exist such as stepping of the adherend, inward or outward tapering of the adherend, and wavy-shaped adherends^28^. Recent experimental work has shown compliance-tailoring of the bondlayer via AM to be a facile and effective approach to improving multilayer performance, wherein increased compliance of the bondlayer in the area of stress concentration reduces the magnitude of stress concentration by diffusing stresses^6,7^. Analytical and numerical studies suggest that compliance-tailoring of the adherends is a possible (but questionably effective) approach for reducing bondlayer stress concentrations^18,19,29–31^, although no study exists where this concept is experimentally implemented due to limitations in conventional manufacturing. As an example, numerical studies of adherend compliance tailoring of advanced composites^18,19^, motivated by the possible (but unrealized) tailoring of adherend modulus by varying the composite braiding angle, showed reductions in critical stresses (shear stress and peel stress reduction of ~20% and 5%, respectively, as measured at the midline of bondlayer), suggesting that adherend-tailoring is a viable approach in improving bonded system performance. Thus, the effect of adherend tailoring on multilayer strength, toughness, and other critical performance metrics are not known. Here, multimaterial jetting AM is utilized to fabricate material- and morphology-tailored adherends. Guided by the numerical models, the influence of adherend-tailoring on multilayer performance is explored numerically, and quantified experimentally for the first time.

## Materials and Methods

### 3D printing and testing of multilayers

Advances in multimaterial additive manufacturing (AM) provide a flexible way of achieving controlled composition of materials in 3D for a variety of applications^6,32–42^. This permits fabrication of structures with spatially-tailored and even customized material properties, *e.g*.,^37,43–49^. In this study, multimaterial jetting 3D printing technology is utilized to fabricate multilayers with spatially material- and morphology-tailored adherends. An Object Connex260 Polyjet 3D printer (Stratasys Ltd., USA) is used to print SLJ multilayers from CAD models created using Solidworks (Dassault Systemes, France). The printer has a resolution of 16 µm in the *z*- direction and 42 µm in the *x*- and *y-*directions. Nozzles on the print heads can jet two types of photosensitive polymers namely VeroWhitePlus^TM^ RGD835 (rigid polymer) and TangoPlus FLX930 (elastomer-like material) in varying proportions. A UV (ultraviolet) laser is used to cure the polymer before the next layer is deposited (on the cured preceding layer) to produce solid structures. The ability of this printer to print and co-cure two photopolymers simultaneously results in tailorable materials termed as digital materials. In order to obtain a desirable surface finish and also to print overhanging structures, a disposable support material named Objet Support SUP705 is printed and later removed by washing with water. The effect of printing direction on the mechanical properties of the 3D printed multilayers, a common and noted concern with 3D Printing^50–56^, is avoided in this study by printing all the samples in the same orientation. The constitutive properties of the different 3D printed polymers are measured using dogbone specimens as discussed in supplementary information section S1, with the linear elastic properties summarized in Fig. S1 and Table S1. The linear elastic modulus of the polymers spans three orders of magnitude, from 1 MPa to over 1 GPa, providing significant freedom for spatial-tailoring. Load transfer in multilayers is greatly dependent on the overlap/bond-length (2*l* in Fig. 1)^12^. The preferred overlap length (when 2*l* ≥ 2*l*_*crit*_, where *l*_*crit*_ is called critical length) is that which enables transfer of the tensile load applied to the adherends predominantly in shear by the bondlayer. Based on Volkersen’s analysis^11^, which considers only axial deformation in the adherends and shear deformation in the bondlayer, 2*l*_*crit*_ for the joint configuration explored in this study is calculated to be ≈400 mm. The overlap length (2*l*) of 50 mm used in the study is a sub-critical bondlength case, consistent with 3D printer build volume limitations. Section S4 contains detailed explanations of calculation of critical bondlength both analytically and via FEA. The various geometries of printed SLJs are described later in the Results section, with an overview of the experimentally-realized SLJs shown in Fig. 1. The SLJs have grip spacers and tabs that are also 3D printed, as discussed in supplementary information section S2 (see Fig. S2).

A Zwick-Roell tensile testing machine with 2.5 kN load cell having an accuracy of <± 0.25% for the measurement range of 10 to 2500 N, and <± 1% for the measurement range of 2.5 to 10 N was used to perform the mechanical testing of the additively manufactured multilayer SLJs. Tensile load was applied in displacement control at a cross head speed of 5 mm/minute, and the displacement was measured with a travel resolution of 0.04 μm at the grips (see Fig. S2). Each case was printed and tested three times to ensure repeatability of the results. 2D strain fields on the sides of the multilayer SLJs were evaluated using digital image correlation (DIC). Random speckle patterns were created on the sides of the specimens by spraying acrylic paints of white and black color using an air brush^57,58^. Images of the specimen during loading were captured at 1 Hz using a 5.0 MP monochrome camera and Vic-Snap Acquisition Software (Version 8, Build 489). 2D strain on the specimen side surface was evaluated by analyzing the captured images using Vic-2D software (version 6.0.6, build 451).

### Finite element analysis

A two-dimensional linear-elastic finite element model was used to analyze the AM-enabled multilayers with Abaqus/Standard FEA Version 6.12. The multilayer SLJ was modeled as a plane-strain problem and geometric parameters used were identical to those used for the experimental study (see Fig. S2) and the elastic properties taken from the dogbone experiments (see Table S1). The models were meshed with an 8-node biquadratic plane-strain quadrilateral element (CPE8). The SLJ multilayers are known to have high stress gradients near the free edges of the bondlayer^59^. A mesh convergence study^60^ indicated that a mesh size below 0.375 mm has negligible effect (less than 2% change in peak stresses with further reduction of mesh size) on the stress distribution in the bondlayer midline. The mesh size used for the bondlayer in this study was 0.15 mm and the same element size was used for the adherends in the 2*l* overlap region. Away from the overlap region, a coarser mesh was adopted for the adherends as shown in Fig. 2a. On the left-hand side, the adherend is constrained in the *x*- and *y*-directions as shown in Fig. 2a to replicate the experimental gripping scenario. A tensile stress σ_*∞*_ is applied to the vertical edge of the adherend on the right-hand side in the *x*-direction as shown in Fig. 2a.

## Results and Discussion

Preliminary numerical analyses and parametric studies were conducted in order to understand the effects of different adherend tailoring schemes on the stress-state in the bondlayer to define tailoring schemes (the tailored adherends are shown in Fig. 1b with modulus profiles shown in Fig. 1a) that minimize peak stresses in the bondlayer. As explained earlier, the adherends are tailored to have increased compliance near the overlap ends to reduce stress concentrations at the overlap ends as shown in Fig. 2. Four different types of isotropic tailoring schemes (as described in section S2) are explored in the preliminary study, namely constant, linear, quadratic, and exponential schemes (see Fig. S3). Different values of modulus ratio *E*_1_/*E*_2_ (where *E*_1_ and *E*_2_ are the maximum and minimum isotropic Young’s moduli of the adherends in the tailored region respectively) and tailored lengths as parameterized by *l*_*t*_/2*l* are considered. The maximum normalized peel and shear stresses in the midline of the bondlayer are shown in Fig. S4 for all four tailoring profiles, modulus ratios *E*_1_/*E*_2_, and tailoring lengths *l*_*t*_. Section S2 contains a detailed discussion of these preliminary FE results from which two key observations are made: (i) bondlayer stress reductions due to isotropic modulus tailoring of the adherends, even for large value of *E*_1_/*E*_2_ =  2000, are very small regardless of the choice of *lt*/2*l* for a sub-critical bondlength of 2*l* = 50 mm used in the experiments. In fact, most of the tailoring schemes studied have opposite intended effect on peel stress σ_*yy*_, *i.e*., the tailoring increases peel stress significantly (see Fig. S5 for the comparison of peak peel and shear stresses of linear tailoring configurations with the baseline, and peak peel stress increases are noted up to 5X); (ii) peel stress increase is because the enhanced compliance of the adherends reduces the bending stiffness at the ends of the overlap significantly, giving rise to higher bondlayer peel stresses. This is an adherend-morphology effect not present in bondlayer tailoring. Note that the net effect of reduced bending moment (see Table S2), and reduction in bending stiffness of the joint, as a result of adherends compliance-tailoring determines the state of stress in the system. This latter observation provided the impetus for investigating anisotropic and morphology-tailored approaches to adherend-tailoring to minimize peel stresses via reduced through-thickness modulus *E*_*y*_, and by constructing an adherend cross-section in a layered/sandwich architecture that allows independent axial (*x*) and transverse (*y*) compliance tailoring, while effectively retaining bending stiffness.

For a better understanding of the effect of anisotropic tailoring of adherends on the bondlayer stresses, numerical studies were performed by tailoring axial and transverse moduli (*E*_*x*_ and *E*_*y*_) independently in a single-step over the graded length, as delineated in section S2. For *E*_*x*_ tailoring, in the tailored region of length *l*_*t*_, *E*_*y*_ = *E*_*z*_ = *E*_1_ and *E*_*x*_ = *E*_2_ (note that for each case, *E*_*x*_ was kept constant in the tailored region) and the analysis was done for different modulus (*E*_1_/*E*_2_ = *E*_1/_*E*_x_) and length (*l*_*t*_/2*l*) ratios. Similarly, analyses were performed for *E*_*y*_ tailoring, in the tailored region of length *l*_*t*_, *E*_*x*_ = *E*_*z*_ = *E*_1_ and *E*_*y*_ = *E*_2_. Peak normalized peel and shear stresses in the midline of the bondlayer for *E*_*x*_ and *E*_*y*_ tailoring shown in Fig. S7 indicate that tailoring the adherend modulus in the *y*-direction (*E*_*y*_) from *E*_1_ to *E*_2_ in a single-step, while keeping *E*_*x*_ = *E*_1_, reduces the peel (maximum of 27% reduction) and shear (slight reduction of ≤4%) stress concentrations in the bondlayer, while tailoring of modulus in the *x*-direction (*E*_*x*_) from *E*_1_ to *E*_2_ has minimal effect on the maximum shear stress, and typically increases the peel stress (see Fig. S7). The reduction of *E*_*x*_ reduces the bending stiffness of the system, which in turn increases the rotation of the joint, leading to increased peel stresses. Implementing anisotropic *E*_*y*_ tailoring practically as done in the numerical studies is not currently possible even with multimaterial jetting AM, therefore, adherends with a layered/sandwich architecture over length *l*t whose core is comprised of a compliant material (constant modulus *E*_2_) are conceived (see Fig. S8). Due to this, the effective longitudinal modulus E^*^_x_ of the adherends are higher than the effective transverse modulus E^*^_y_ in the tailored region, effectively achieving anisotropic tailoring. This helps to maintain the bending stiffness while minimizing the transverse stiffness mismatch. Numerical analyses were performed on multilayer SLJs with layered/sandwich architectured adherends (geometric details shown in Fig. S8) for different values of *E*_1_/*E*_2_ and *l*_*t*_/2*l*. The results indicate that an ~45% reduction in peel stress can be realized for *l*_*t*_/2*l* = 0.3, 0.8, and 1.0, while the reduction in shear stress is minimal (<3%) (see Fig. S9). From the highest performing cases, in terms of maximum stress reduction, *l*_*t*_/2*l* = 0.3 with *E*_1_/*E*_2_ ≈ 3300 is explored experimentally because it has the lowest length of tailoring which reduces the propensity for adherend failure and multilayer SLJ stiffness reduction.

The monotonic tailoring configuration is realized via AM by applying a gradual variation of modulus from *E*_1_ to *E*_2_ in the beginning of tailored region (*x* = ± *l* − *l*_*t*_). This increases the *l*_*t*_/2*l* ratio from 0.3 to 0.35 (see discussion in section S2). This configuration is denoted as ‘monotonic tailoring’ (see Fig. 1). To further improve on the monotonic tailoring, we employ a smoothly increasing modulus near the overlap ends in the tailored region to an intermediate value E_3_ (*E*_2_ < *E*_3_ < *E*_1_) to maintain high bending stiffness at the critical end region (see Fig. S10). This configuration is denoted as ‘non-monotonic tailoring’ and is also realized experimentally (see Fig. 1). The non-tailored portion of the adherends are printed using VeroWhitePlus^TM^ RGD835 (VW) having a Young’s modulus (*E*_1_) of 2085 MPa and the bondlayer is printed using Shore40 (S40) having a Young’s modulus (*E*_*a*_) of 1.02 MPa. The Young’s modulus in the tailored region varies between *E*_1_ and *E*_2_ and is achieved by making use of different digital materials (see discussion and results in Section S1, and Fig. S8 for geometry of the tailored SLJ multilayers). The modulus variation in the tailored region of length *l*_*t*_ is shown in Fig. 1a.

**Figure 3:**
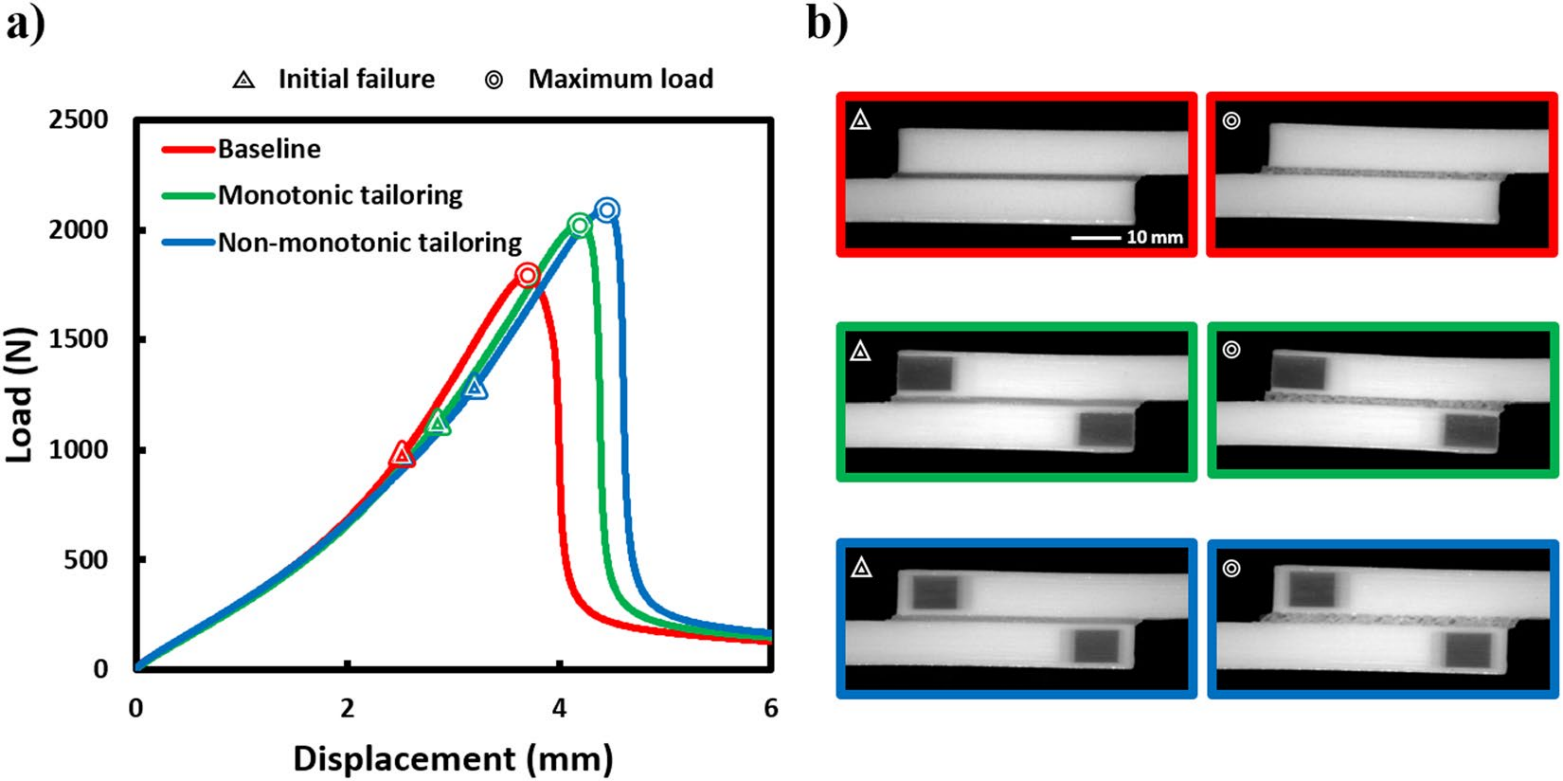
Performance comparison of additively manufactured SLJ multilayers with baseline and tailored adherends: (**a**) Representative load-displacment behaviour. Points of failure initiation and maximum load indicated. (**b**) Side view Optical images of representative specimens near to, but before, failure initiation and final failure.

**Figure 4:**
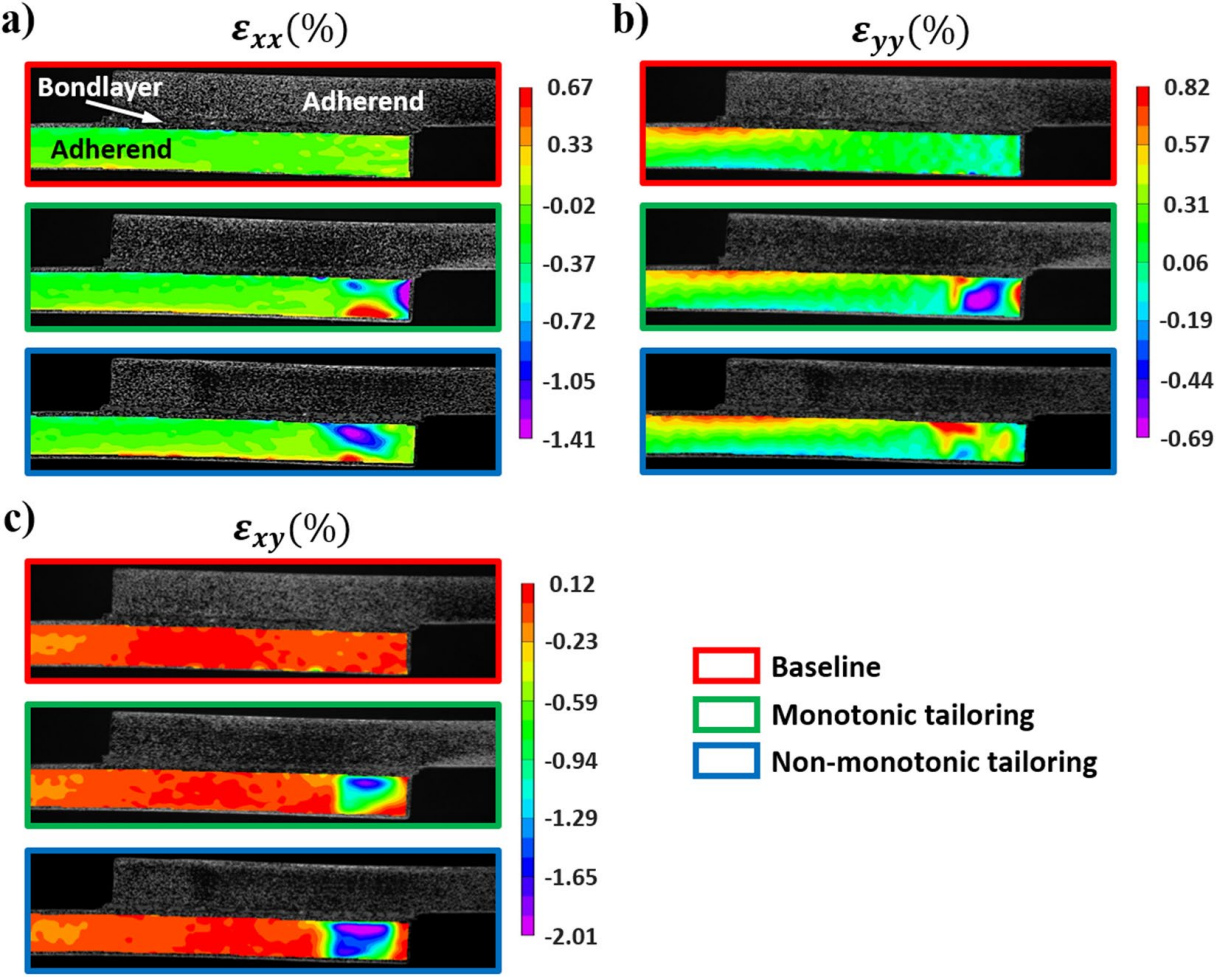
2D full-field strain distrbution in the bottom adherends of tailored and baseline cases under a tensile load of 1520 N: (**a**) axial strain, (**b**) peel strain, and (**c**) shear strain.

Figure 3a shows representative load-displacement curves for the baseline and tailored (monotonic- and non-monotonic) multilayers tested, with side view optical images of the samples near to, but before, failure initiation and final failure as shown in Fig. 3b. The points corresponding to failure initiation and final failure are denoted in the load-displacement curves. The synchronized macroscopic load-displacement response for the three configurations with their corresponding deformations as a function of load can be seen in the supplementary video SV1. Optical strain mapping near failure is shown for all three configurations in Fig. 4, clearly showing strain redistribution at the free ends of the adherends for both tailored configurations. Note that in monotonic tailoring, strain concentration occurs near the free ends of the adherends because the adherend free ends are compliant, whereas in the case of non-monotonic tailoring, the strain concentration occurs slightly away from the free ends. Taken together, the experiments indicate that the stress and strain redistribution in the bondlayer due to material- and morphology-tailoring of the adherends results in improved performance of the SLJ multilayers as summarized in Table 1. Both tailoring schemes resulted in an insignificant change in joint stiffness and have positively affected the strength (13% improvement for monotonic tailoring and 20% for non-monotonic tailoring) and maximum deflection before final failure (12% improvement for monotonic tailoring and 18% for non-monotonic tailoring). Increases in strength and maximum displacement before final failure have led to a significant enhancement in toughness of the tailored systems (especially, non-monotonic tailoring with a toughness enhancement of 48% compared to the baseline case). These enhancements in performance of tailored SLJs without any reduction in joint stiffness demonstrates significantly improved multilayer joints via adherend tailoring.

**Table 1:** Summary of experimental performance of single lap joint multilayers: statistically significant changes compared to baseline case are shown in bold.

Design Configuration	Joint stiffness (N/mm)	Maximum load (N)	Deflection at break (mm)	Toughness (N*mm)
Baseline	297.11 ± 3.5	1785.82 ± 58.84	3.74 ± 0.08	2756.48 ± 175.37
Monotonic tailoring	300.35 ± 5.59	2070.51 ± 60.94 **(+13%)**	4.19 ± 0.09 **(+12%)**	3633.99 ± 179.27 **(+29%)**
Non-monotonic tailoring	306.39 ± 6.34	2146.52 ± 32.87 **(+20%)**	4.41 ± 0.11 **(+18%)**	4088.39 ± 285.11 **(+48%)**

In order to have a better understanding of the change in the load transfer mechanism in the SLJ multilayers with tailored adherends compared to the baseline case, finite element analyses of all experimentally realized sub-critical cases were performed. Stress distributions in the midline of the bondlayer are plotted in Fig. 2b. Although there is no clear difference between the shear stress distributions for the different cases, the peel stress distribution for both tailored cases has changed substantially from the baseline. The stresses in the bondlayers of the tailored cases are redistributed in such a way that the peak peel stresses are reduced as summarized in Table 2, showing a maximum peel stress reduction of 47% for monotonic tailoring and 57% for non-monotonic tailoring. Thus, stress redistribution in the bondlayer due to material- and morphology-tailoring of the adherends, favorably affects the performance of the multilayer SLJ systems, in agreement with the experimental performance improvements (notably strength).

**Table 2:** Summary of stress analysis in the SLJ multilayer bondlayer for the tailored and baseline adherend configurations. Significant changes compared to baseline case are shown in bold.

Design Configuration	Maximum peel stress (MPa)	Maximum shear stress (MPa)
Baseline	0.147	0.215
Monotonic tailoring	0.078 **(-47%)**	0.213
Non-monotonic tailoring	0.064 **(-57%)**	0.0.214

**Figure 5:**
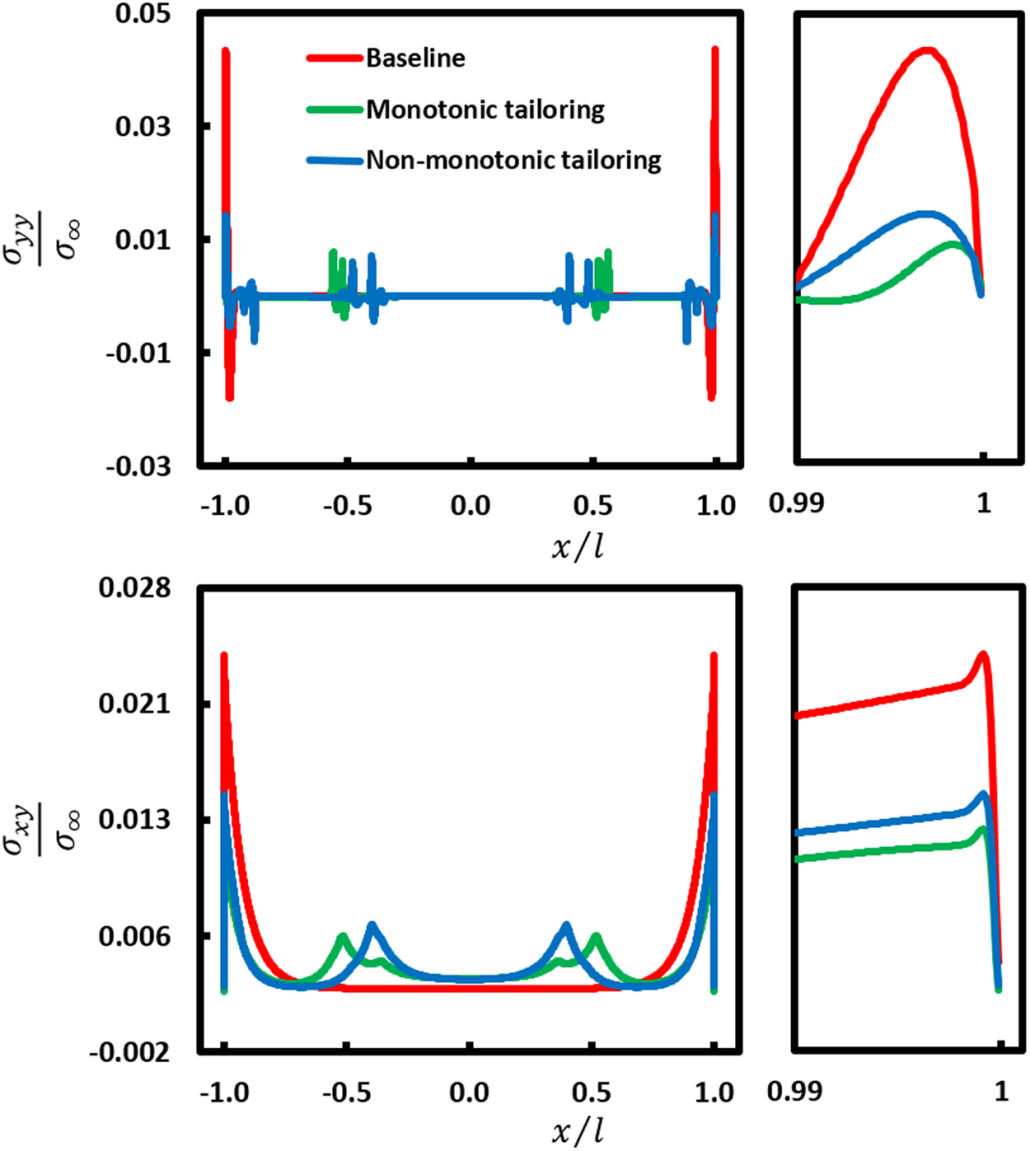
Stress analysis of the mutlilayer SLJs at critical bondlength (2*l* = 4m$$)$$ using FEA: Peel and shear stress distribtution in the midline of the bondlayer over the bondlength for baseline and tailored cases under a tensile load of 200 N. Right side plots show a zoomed in view of the stress peaks (note that right side plots have the same $$y$$ axis scale as the corresponding left side plots).

In order to understand the effect of adherend tailoring on the load transfer through the bondlayer in the SLJ multilayers with a longer, critical, overlap length, FE analyses were repeated with 2*l* ≈ 2*l*_*crit*_ = 4 m as shown in Fig. 5. It can be observed that, compared to the sub-critical overlap case (2 *l* < 2*l*_*crit*_), both peak peel and shear stresses have reduced significantly and both peaks have shifted towards the bondlayer free edges as expected. Table 3 summarizes the maximum peel and shear stresses of tailored and baseline cases. Maximum peel and shear stresses for the tailored cases are reduced over 67% and 38%, respectively. These reductions are higher than those obtained for the sub-critical tailored cases. Unlike the sub-critical overlap case, there is significant reduction of maximum stresses for both tailoring schemes due to higher bending moment reduction in tailored configurations compared to the baseline due to increased compliance of the adherend ends for the same *l*_*t*_/2*l* = 0.3 (see Table S3 and related discussion). Note that higher peak stress reductions are observed for monotonic tailoring vs. non-monotonic tailoring in the longer critical overlap length, an opposite trend to the sub-critical length case. At the longer overlap length, the monotonic tailoring has reduced bending moment due to increased compliance relative to the non-monotonic case, while the bending stiffnesses at the ends of the overlap remain unchanged for both cases with increased bondlength. It should be noted that the sub-critical length SLJ multilayer configuration has greater practical importance in engineering applications than the critical length case.

**Table 3:** Summary of the stress analysis of SLJ multilayer for 2*l* ≈ 2*l*_*crit*_: Significant changes compared to baseline case are shown in bold.

Design Configuration	Maximum peel stress (MPa)	Maximum shear stress (MPa)
Baseline	0.044	0.0236
Monotonic tailoring	0.008 **(−80%)**	0.0123 **(−48%)**
Non-monotonic tailoring	0.014 **(−67%)**	0.0149 **(−38%)**

## Conclusions

For the first time, multilayer joints with compliance- and morphology-tailored adherends are realized by utilizing multimaterial jetting additive manufacturing, and the structural performance is evaluated experimentally. Numerical stress analyses indicate that the effect of tailoring of adherends on the bending stiffness and bending moment in the bonded multilayer needs to be considered especially when the bondlength is subcritical (lower than the critical length required for shear-dominated load transfer, and of primary design importance) as in this study. Reduction in bending stiffness of the multilayer system with increased adherend compliance (isotropic modulus tailoring) increases peel stresses in the bondlayer, obviating positive intended effects of straightforward adherend compliance tailoring, and is a key difference between bondlayer/adhesive vs. adherend tailoring, *i.e*., adherend tailoring requires both material- and morphology-tailoring. In addition to anisotropic tailoring, multilayers with layered/sandwich architectured adherends were developed that enable a higher bending stiffness at the tailored overlap ends resulting in lower peak peel stresses in the bondlayers. Tailored layered/sandwich architectured adherends (as a result of reduced bondlayer stresses) compared to the baseline configuration shows significant increases in tested mechanical joint metrics with negligible effect on the joint stiffness. Numerical studies on the multilayers with monotonically-tailored and layered/sandwich architecture adherends at critical bondlengths (bondlength sufficient enough for shear dominated load transfer) show that the reduction in peak stresses are relatively larger for systems with critical bondlengths than for the subcritical bondlength systems, underscoring the broad impact of the adherend tailoring approach. Spatial tailoring of adherends’ compliance and morphology demonstrated in this study adds to the techniques available to increase the mechanical performance of bonded multilayers. Combined with advances in printing continuous^61,62^ and discontinuous^32,63^ fiber reinforced structural materials, the tailoring strategies introduced here can more fully exploit AM for structural applications. Recent work that establishes compliance tailoring for a printed structural polymer reinforced with discontinuous structural fibers is a facile way to obtain compliance tailoring in AM printed structural components^44^. Spatial tailoring of the adherends compliance is a viable approach to enhance the structural performance of multilayers both at subcritical and critical bondlengths, with particular attention needing to be paid to engineering the bending stiffness of the adherends appropriately so as not to adversely induce high peel stresses.

## Electronic supplementary material

Supplementary Information

Supplementary video
