# Supplementary material for: Strength and Performance Enhancement of Multilayers by Spatial Tailoring of Adherend Compliance and Morphology via Multimaterial Jetting Additive Manufacturing
Source: Sci Rep. 2018 Sep 11;8:13592. doi: 10.1038/s41598-018-31819-2 (PMC6134147; doi:10.1038/s41598-018-31819-2)
Supplement: Supplementary file 1 — Supplementary Information [file 41598_2018_31819_MOESM1_ESM.docx]

**Supplementary information**

**Strength and Performance Enhancement of Multilayers by Spatial Tailoring of Adherend Compliance and Morphology via Multimaterial Jetting Additive Manufacturing**

Jabir Ubaid^†^, Brian L. Wardle^‡^, S. Kumar*^,†^

^†^Department of Mechanical and Materials Engineering, Khalifa University of Science and Technology, Masdar Institute, Abu Dhabi 54224, UAE

^‡^Department of Aeronautics and Astronautics, Massachusetts Institute of Technology, Cambridge, MA 02139, USA

* Corresponding author. Fax: +971 2 8109901.

Email address: s.kumar@eng.oxon.org (S. Kumar)

# **S1. Materials characterization**

Tensile tests on different materials printed using the Object Connex260 3D printer were carried out in order to assess the linear elastic properties as per ASTM D412^1^. Dogbone samples with gauge length of 25mm, width of 6mm, and thickness of 2 mm were printed using the 3D printer. A random white or black speckle pattern was applied to the sample surface by spraying acrylic paints using an air brush for digital image correlation analysis ^2,3^. The samples were tested under uniaxial tensile load using a Zwick-Roell universal testing machine with a constant crosshead speed of 5mm/minute and images of the surface captured while the specimen was being loaded using a CCD camera of 5.0 MP at 2 Hz. A 2.5 kN load cell was used to measure the load, $F$ during the test from which engineering stress, $\sigma_{xx-eng}$ was calculated such that $\sigma_{xx-eng}= F/{A_{0}}$ where, $A_{0}$ is the original cross-sectional area. Digital image correlation was performed using VIC-2D software on the captured images of the samples in order to find the engineering surface strain in the loading direction ($\varepsilon_{xx-eng}$) and in lateral direction ($\varepsilon_{yy-eng}$). The average strain is measured at an area within the gauge length of the sample over a width that is slightly less than the specimen width so as to reduce the error in strain calculation at the boundaries to evaluate the average of strain components ($\varepsilon_{xx-eng}$ and $\varepsilon_{yy-eng}$) ^4^. Representative engineering stress - engineering strain response curves for the different materials are shown in Figure S1. Elastic moduli for all the samples are determined by calculating the initial slope of the $\sigma_{xx-eng}$ – $\varepsilon_{xx-eng}$ curves. Poisson’s ratio ($\nu$) for all the samples are found by calculating the initial slope of $\varepsilon_{yy-eng}$vs. $\varepsilon_{xx-eng}$ curves. The material properties determined are summarized in Table S1 based on 3 repeat specimens for each material.

**Figure S1** Representative engineering stress – engineering strain response of 3D printed materials used in this study to achieve tailored adherends: Curves of stiffer materials on the left hand side and softer materials on the right hand side.

**Table S1** Linear elastic properties of 3D printed polymers used in this study.

| **Material Name** | **Young’s Modulus (MPa)** | **Poisson’s Ratio** |
| --- | --- | --- |
| VeroWhite ($E_{1}$) | 2,085 | 0.340 |
| TangoPlus ($E_{2}$) | 0.63 | 0.495 |
| Shore40 ($E_{b}$) | 1.02 | 0.493 |
| Shore50 | 1.42 | 0.491 |
| Shore60 | 2.50 | 0.490 |
| Shore70 | 5.02 | 0.480 |
| Shore85 | 7.90 | 0.471 |
| Shore95 | 46.0 | 0.440 |
| DM8430 ($E_{3}$) | 1,045 | 0.390 |
| DM8425 | 1,556 | 0.380 |

# **S2. Preliminary finite element analysis of bondlayer stresses with adherend tailoring**

**Figure S2** Geometric configuration of multilayer SLJs used for preliminary FE study. Geometric properties are the same as in the experiments and the overlap length 2l is sub-critical (see discussion in main text). $2l=50 mm$,$L=38 mm$, $h=9 mm$, $t=1.5 mm,$ $w=25 mm$, and the length of the tailored region, $l_{t}=15mm$ in this figure.

Preliminary numerical studies via two-dimensional linear elastic finite element analysis using Abaqus/Standard FEA Version 6.12 were carried out in order to identify trends and develop suitable schemes of adherend tailoring. Geometric details of the single lap joint used for the preliminary stress analysis study are shown in Figure S2 and are chosen to represent typical single-lap joint configurations (e.g., adherend vs. bondlayer relative thickness and stiffness). The modulus of the baseline/untailored adherend is $E_{1}$ and the bondlayer is$E_{b}$. The four adherend compliance profiles (modulus profiles) considered in this preliminary study (where$E_{2} < E_{1}$) are: (i) step variation (constant modulus, $E_{2}$ over the tailored region), (ii) linear variation, (iii) quadratic variation, and (iv) exponential variation where the modulus reduces from $E_{1}$ to $E_{2}$ at the free ends of the adherends (at $x= \pm l$). Analyses were performed for all the four modulus profiles for different length ratios${l_{t}}/{2l}$, and different modulus ratios ${E_{1}}/{E_{2}}$,. The four adherend compliance profiles for ${l_{t}}/{2l}=0.6$ and ${E_{1}}/{E_{2}=2000}$ are plotted in Figure S3 together with equations used for the modulus variation in the tailored region. The focus of the analyses is determining the peel and shear stress values in the bondllayer, and evaluating these along the midline, as a criteria for determining the effectiveness of the different tailoring schemes, *i.e.,* these maximum peel and shear stresses along the bondlayer centerline are used as criteria to determine the effect of varying ${l_{t}}/{2l}$ for the four adherend compliance profiles. Note that both peel and shear stresses generally concentrate at the free edges of the adherend, as shown in Figure 2 of the main text.

**Figure S3** Upper adherend (see Figure S2) modulus profiles for step, linear, quadratic and exponential adherend tailoring schemes (exemplary$l_{t}/2l = 0.6$, and ${E_{1}}/{E_{2}}=2000$) used in preliminary numerical modeling studies. Note that both the upper and lower adherends are tailored in each case, as shown in Figure S2).

The multilayer SLJ in Figure S2 is an example of a joint design which has a sub-critical overlap length, *i.e.,* the length at which the bondlayer shear stress at the mid-bondlength (at $x=0$) does not go to zero. Such sub-critical joints are of engineering interest as in many design situations, and particularly for the SLJ configuration, minimizing the length over which the load is transferred from one adherend to the other is a design constraint or objective. The sub-critical joint dimensions in Figure S2 correspond to the experimental cases realized on the 3D printer. Longer joints that are beyond the critical length, and beyond the build volume of the 3D printer, are analyzed and discussed in the main text in the Results section. The distribution of stresses for the baseline case in Figure 2b of the main text show the key features of the bondlayer stress distribution: both shear and peel stresses have maxima near the free ends of the bondlayer (at $x=\pm l$). Increasing adherend compliance in this region is expected to reduce these maxima by reducing the mismatch in moduli driving the stresses, similar to increasing compliance of the bondlayer in this region, but also by reducing the bending moment on the system that arises from a reduction in eccentricity of load in tailored configurations. The maximum normalized peel and shear stresses in the midline of the bondlayer are shown in Figure S4 for all four tailoring profiles, modulus ratios${E_{1}}/{E_{2}}$, and tailoring lengths $l_{t}$. The tailoring, even when considering extreme changes in compliance of 2000X and over any $l_{t}/2l$ length ratio, has very little positive effect on the maximum stresses. At best the results are mixed (as in the linear tailoring series in Figure S4b) where there is an increase in peel stress $\sigma_{yy}$with a (very small) decrease in shear stress $\sigma_{xy}$in the bondlayer, and at worst, the largest effect (see Figure S4a) is an increase in peel stress from $\approx$ 13% to over 65% of $\sigma_{\infty}$with smaller but also significant increases in maximum shear stress. The other adherend tailoring schemes have mixed, but small ($<$ 5%), changes in stresses.

The series of results in Figure S4b for linear isotropic tailoring is the most instructive in explaining these findings as the sharp transition of modulus in the step tailoring cases of Figure S4a makes the comparison of maxima less clear. In Figure S4b for linear tailoring, the peel stress ($\sigma_{yy}$) is noted to increase (by ~20%), with an even smaller decrease in maximum shear stress. The peel stress increases due to isotropic compliance tailoring of adherends can be understood by considering the stress distributions in the bondlayer midline for linear tailoring cases (${l_{t}}/{2l}$ of 0.2, 0.6 and 1.0 with ${E_{1}}/{E_{2}=2000}$) shown in Figure S5; the linear tailored configurations have slightly reduced shear stress concentrations at the ends, but higher peel stress relative to the baseline configuration, with a small shift outward of the location of the peak peel stress as the length of tailoring increases. Increased compliance at the ends of the adherends, as demonstrated by the linear-tailoring, has the positive effect of reducing the bending moment in the multilayer SLJ , but a negative net effect on peel stress due to reduced adherend bending stiffness. So, while adherend tailoring using increased compliance towards the free end can have small positive effects on shear stress maxima, it has a net negative effect on peel stress maxima as shown in Figure S4. Thus, increased peel stress due to reduced bending stiffness, particularly at the free edges of the adherends, is a critical consideration in adherend tailoring that is not operative in bondlayer compliance tailoring because the bondlayer is symmetric about the multilayer midline.

**Figure S4** Peak peel and shear stresses at the bondlayer midline for different length ratios (${l_{t}}/{2l}$) vs modulus ratio (${E_{1}}/{E_{2}}$) for different adherend tailoring configurations: (a) Step tailoring with modulus, $E_{2}$ over the tailored region, (b) linear, (c) quadratic, and (d) exponential variation from $E_{1}$to $E_{2}$ towards adherend free end. Note that the baseline case (${l_{t}}/{2l}=0$) is a horizontal line on these plots.

**Figure S5** Peak peel and shear stresses at the bondlayer midline for different length ratios (${l_{t}}/{2l}$) for linear tailoring configuration (${E_{1}}/{E_{2}}=2000$).

To explore the interplay between reduced bending moment and bending stiffness due to increased compliance in the adherends at the overlap ends, bending stiffness in the overlap region of the SLJ multilayer is plotted in Figure S6 and bending moment at the ends of overlap region is tabulated in Table S2 for the baseline and SLJ multilayers with linear compliance tailored adherends (${E_{1}}/{E_{2}=2000}$). Calculation of bending stiffness and bending moment is straightforward and further explained in section S5. These examples show that the linear tailored configurations have less than 4% reduction (even when ${l_{t}}/{2l}=1.0$ and ${E_{1}}/{E_{2}=2000}$) in bending moment at the overlap ends for the sub-critical bondlength considered here (a positive effect from peel stress perspective), accompanied by a bending stiffness reduction of $\approx$ 90 % at the overlap ends (a negative effect from the peel stress perspective) which is the cause of the net increase in peel stress in the bondlayer.

**Table S2** Bending moment per unit width at the overlap ends for SLJ multilayers with linear compliance tailored adherends (${E_{1}}/{E_{2}=2000}$) vis-à-vis the baseline configuration for sub-critical bondlength of $2l = 50$ mm.

| **Design Configuration** | **Bending moment (**$\boldsymbol{N*m}/\boldsymbol{m}$**)** |
| --- | --- |
| Baseline | 24.34 |
| Linear tailoring, ${l_{t}}/{2l}=0.2$ | 23.99 |
| Linear tailoring, ${l_{t}}/{2l}=0.6$ | 23.98 |
| Linear tailoring, ${l_{t}}/{2l}=1.0$ | 23.45 |

Two concepts are developed in this work to address the observations discussed above, particularly the net negative effect of reduced adherend bending stiffness on the maximum peel stress in the multilayer SLJs: (i) anisotropic compliance tailoring of the adherends, and (ii) utilizing a layered/sandwich adherend architecture. The anisotropic tailoring is explored via numerical studies to tailor the longitudinal and transverse moduli ($E_{x}$ and $E_{y}$) independently as step tailoring. Taking the material properties to be transversely isotropic for the geometry shown in Figure S2, for $E_{x}$ tailoring, in the tailored region of length$l_{t}$, $E_{y}= E_{z}= E_{1}$ and $E_{x}= E_{2}$ (note that for each case, $E_{x}$ was kept constant in the tailored region) and the analysis was done for different modulus (${E_{1}}/{E_{2}}={E_{1}}/{E_{x}}$) and length (${l_{t}}/{2l}$) ratios. Similarly, analyses were performed for $E_{y}$ tailoring, in the tailored region of length$l_{t}$, $E_{x}= E_{z}= E_{1}$ and $E_{y}= E_{2}$ . Peak normalized peel and shear stresses in the midline of the bondlayer for $E_{x}$ and $E_{y}$ anisotropic tailoring are shown in Figure S7 and can be compared to Figure S4a. The plots indicate that for $E_{x}$ tailoring, there is no positive effect on peel stresses in the bondlayer, while there is slight decrease in peak shear stresses ($<$7%), consistent with the prior discussion. This is in contrast to $E_{y}$ tailoring, which has a considerable positive effect on peel stresses and a slight positive effect on shear stresses in the bondlayer, compared to the baseline configuration. For ${l_{t}}/{2l}\geq0.2$ and${E_{1}}/{E_{2}}\geq500$ there exists a minimum of 12 % and a maximum of 27% reduction of peak peel stresses, while the highest reduction of peak shear stress obtained is less than 4%. These findings indicate that tailoring of the adherend modulus anisotropically, particularly in the $y$-direction ($E_{y}$), reduces the peel stress concentration in the bondlayer whereas tailoring of modulus in $x$-direction $(E_{x})$ has adverse effects on peak stresses in the bondlayer because of the reduction in bending stiffness noted earlier. Reducing $E_{y}$ (only) will not significantly affect the rotation of the joint, but will lower through-thickness (transverse) stiffness directly causing redistribution of stresses in the bondlayer, notably reducing the peak peel stresses.


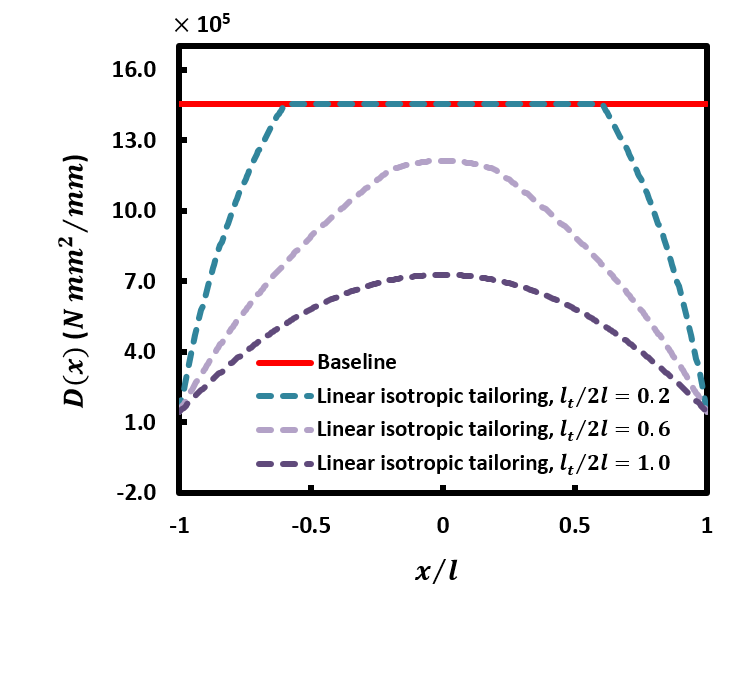


**Figure S6** Bending stiffness variation in the overlap region for linear compliance tailored SLJs (see Figure S3) vis-à-vis the baseline configuration.

Since, $E_{x}$and$E_{y}$ tailoring as done in the numerical study is difficult to achieve practically, even with the functionality of AM, adherends with a layered/sandwich morphology (or architecture) are conceived that allow anisotropic tailoring (via effective modulus) and that retain bending stiffness to a significant degree. These layered/sandwich adherend morphologies have a core that is tailored with a compliant material (constant isotropic modulus$E_{2}$) with external layers of isotropic modulus $E_{1}$ with thickness $h_{t}$ and $h_{b}$ over length $l_{t}$as shown in Figure S8. Due to this, the effective longitudinal modulus $E_{x}^{*}$of the adherends are higher than the effective transverse modulus $E_{y}^{*}$in the tailored region, effectively achieving anisotropic tailoring. The effective Young’s moduli of the layered/sandwich architectured adherends are calculated as explained in section S3 and show that with ${E_{1}}/{E_{2}=2000}$ (a step variation), the effective moduli are $E_{x}^{*}=464 MPa$ and $E_{y}^{*}=1.34 MPa$ with$E_{2}=1.04 MPa$. This gives a highly anisotropic set of effective moduli with an anisotropy ratio of 346, and a bending stiffness at the overlap ends (for${l_{t}}/{2l}<1$) that is 41% of the baseline. Numerical stress analyses were performed for different length ratios${l_{t}}/{2l}$ and different moduli ratios${E_{1}}/{E_{2}}$ for the same sub-critical bondlength of $2l =$ 50 mm as before. Figure S9 shows the maximum normalized peel and shear stresses in the midline of the bondlayer layer for SLJs with sandwich architectured adherends. It can be observed that peel stresses are reduced for the cases where ${l_{t}}/{2l}\geq0.15$ and the highest peel stress reduction ($\approx45\%$ reduction) is obtained for ${l_{t}}/{2l}=0.8, {l_{t}}/{2l}=1.0$ and${l_{t}}/{2l}=0.3$. Shear stress peaks are shown to have slight reduction (< 3%) compared to the baseline configuration. These findings indicate that the lower modulus obtained for effective modulus $E_{y}^{*}$compared to $E_{x}^{*}$results in considerably greater reduction of peel stresses in the bondlayer. This is in line with the findings of the numerical results discussed above wherein $E_{x}$ tailoring was found to have adverse, and $E_{y}$ tailoring was found to have positive, effects on the peel stress peaks in the bondlayer. Note that analyses were performed for moduli mismatch ${E_{1}}/{E_{2}}$ as high as $3300$ (a value attainable via the AM experiments utilized herein), allowing the modulus of the core ($E_{2}=0.63 MPa$) to be less than that of the bondlayer ($E_{b}=1.02 MPa$). The results of step tailoring shown in Figure S9 are consistent with the design objective of reducing both peel and shear stress maxima in the bondlayer, but require anisotropic tailoring (either directly, or by utilizing a layered/sandwich adherend tailoring approach. Note the significant reductions of peel and shear stress are obtained at a modulus ratio ${E_{1}}/{E_{2}}$ of several hundred.

From the findings of the numerical studies performed on SLJ multilayers with layered/sandwich architectured adherends, configurations where ${l_{g}}/{2l}=0.3$ and ${E_{1}}/{E_{2}}\approx3300$ were selected for the AM configuration to perform mechanical testing. Among the cases where the greatest peel stress reduction is obtained (${l_{t}}/{2l}=0.3, 0.8,$ and $1.0), {l_{t}}/{2l}=0.3$ is chosen in order to reduce the propensity for adherend failure because larger $l_{t}$ reduces the stiffness of the multilayer and strength of the adherends. In order to avoid the stress concentrations arising from a step jump in modulus at the beginning of the tailored region ($x=l-l_{t}$), a linear variation from $E_{1}$ to $E_{2}$ over a small region that increases ${l_{t}}/{2l}$ from 0.3 to 0.35 is adopted and this configuration is termed monotonic tailoring in the main text (see Figure 1 and related discussion). Layered/sandwich architectured adherends also ensure a consistent adhesion energy as the adherends are not tailored at the adherend-bondlayer interfaces ^5,6^. Bending stiffness of the system, especially near the overlap ends, plays a major role in bondlayer failure by affecting the peel stress at the overlap ends as explained earlier in this section. To increase the bending stiffness at the overlap ends, an additional configuration termed non-monotonic tailoring is also adopted, which is a modified version of the monotonic tailoring scheme, where the modulus in the tailored region close to the overlap ends is smoothly increased to an intermediate value$E_{3}$. Schematics of the tailored configuration, images of 3D printed samples and plots of moduli variation in the adherends are shown in Figure 1 of the main text. Bending stiffness of the baseline configuration and the two architected configurations (monotonic and non-monotonic) from the main text, together with a case of linear tailoring (without sandwich, ${l_{t}}/{2l}=0.3$ and ${E_{1}}/{E_{2}}=2000$) are shown in Figure S10. Compared to linear tailoring, bending stiffnesses are higher for the layered/sandwich architectured configurations at the overlap ends. Further, bending stiffness at the critical overlap ends for non-monotonic tailoring is higher than that of monotonic tailoring as expected. Bending moment at the overlap ends of the baseline configuration and for the two layered/sandwich architectured configurations (monotonic and non-monotonic), together with the case of linear tailoring for $2l\ll{2l}_{crit}$and$2l\approx{2l}_{crit} (\approx4m)$, are tabulated in Table S3. Calculation of $2l_{crit}$ is explained in section S4. Note that ${l_{t}}/{2l}$ is kept at 0.35 for tailored configurations in the cases of $2l\approx{2l}_{crit}$ as in the subcritical case. This means that length of tailoring ($l_{t}$) is higher for 2$l\approx{2l}_{crit}$. These analyses indicate that there is a slight bending moment reduction for all the tailored configurations for the subcritical case, whereas a large reduction ($\geq60\%$) is noted when $2l\approx{2l}_{crit}$ relative to baseline configuration. The higher reduction of bending moment when $2l\approx{2l}_{crit}$ is due to reduced load-eccentricity as a result of increased compliance compared to subcritical case.

**Figure S7** Peak peel and shear stresses at the bondlayer midline for different length ratios (${l_{t}}/{2l}$) vs modulus ratio (${E_{1}}/{E_{2}}$) for anisotropic (only) step adherend tailoring: (a) $E_{x}$ tailoring (${E_{1}}/{E_{2}}={E_{1}}/{E_{x}}$) and (b) $E_{y}$ tailoring (${E_{1}}/{E_{2}}={E_{1}}/{E_{y}}$). Note that the baseline case (${l_{t}}/{2l}=0$) is a horizontal line on these plots. These plots compare to Fig. S4a isotropic step tailoring.

**Figure S8** Geometric configuration of multilayer SLJs with tailored and layered/sandwich architectured adherends. The overlap length $2l$is sub-critical. $h_{c}$ is the height of the core of tailored region, $h_{t}$ and $h_{b}$ are the height of the upper and lower regions of layered/sandwich architecture. $2l=50 mm$,$L=38 mm$, $h=9 mm$, $t=1.5 mm$, $w=25 mm$, $h_{c}=7 mm$, $h_{t}=h_{b}=1 mm$ and, $l_{t}=17.5 mm$ in this figure.

# **S3 Calculation of effective moduli of tailored adherends**

**Figure S9** Peak peel and shear stresses at the bondlayer midline for different length ratios (${l_{t}}/{2l}$) vs modulus ratio (${E_{1}}/{E_{2}}$) of multilayer SLJs with tailored and layered/sandwich architectured adherends: (a) Peak peel stresses and (b) peek shear stresses.

Effective moduli $E_{x}^{*}(x)$ and $E_{y}^{*}(x)$of the individual tailored adherends are calculated using the Voigt and Reuss models ^7-9^, commonly known as ‘springs-in-parallel’ and ‘springs-in-series’, giving:

$$E_{x}^{*} = V_{t+b}E_{1}+V_{ct}E_{ct}$$

$$1/{E_{y}^{*}}={V_{t+b}}/{E_{1}+{V_{ct}}/{E_{ct}}}$$

where$V_{t+b}$ and $E_{1}$ are the volume fraction and Young’s modulus of the upper and lower regions of the layered/sandwich region and $V_{ct}$ and $E_{ct}(x)$ are the volume fraction and Young’s modulus in the core of tailored region.

# **S4 Calculation of critical length**

Critical overlap length, $2l_{crit}$, corresponds to a bondlength that enables the bondlayer to transfer load dominantly in shear and is calculated using Volkersen’s ^10^ analysis, assuming extension-only deformation of adherends and pure shear deformation of the bondlayer:

$$l_{crit}=\sqrt{\frac{E_{1}ht}{2G_{b}}}$$

where $E_{1}$ and $h$ are the (isotropic) Young’s modulus and thickness of the non-tailored adherend respectively, and $t$and$G_{b}$ are the thickness and shear modulus of the bondlayer. The case analyzed is for the stiff ($E_{1}$) adherends so it should be noted that ${2l}_{crit}$ is not conservative relative to tailoring. ${2l}_{crit}$ is calculated to be $\approx$0.4 m using Volkersen’s analysis. As Volkersen’s analysis considers only tensile deformation of the adherends and shear deformation of the bondlayer, $2l_{crit}$ is also calculated using FEA by finding the overlap length at which shear stress saturates near zero at the mid-bondlength in the bondlayer. Using FEA, ${2l}_{crit}$ is calculated to be $>4 m$, an order of magnitude higher than ${2l}_{crit}$ from Volkersen’s analysis, as the bending deformation is captured in the FEA. In this study, ${2l}_{crit}$ is considered to be 4 m.

# **S5 Calculation of bending stiffness and bending moment**

Considering simple bending, the plate bending stiffness per unit width, $D_{ol}(x)$, in the overlap region (see figure S8) is calculated via a simple summation of contributions from the two adherends and the bondlayer:

$$D_{ol}=\frac{E_{xt}^{*}(x)}{1-v_{1}^{2}}\left( \frac{h^{3}}{12}+h\left( \frac{h+t}{2}-d\left( x \right) \right)^{2} \right)+\frac{E_{xb}^{*}(x)}{1-v_{1}^{2}}\left( \frac{h^{3}}{12}+h\left( \frac{h+t}{2}+d\left( x \right) \right)^{2} \right)+\frac{E_{b}}{1-v_{b}^{2}}\left( \frac{t^{3}}{12} \right)$$

where $E_{xt}^{*}$, $E_{xb}^{*}$, $v_{1}$, $E_{b}$, $v_{b}$, $h$, $t$ and $d(x)$ are the effective modulus of top adherend in $x$ direction, effective modulus of bottom adherend in $x$ direction, Poisson’s ratio of adherends, Young’s modulus of the bondlayer, Poisson’s ratio of bondlayer, adherend height, bondlayer thickness, and neutral axis position from the mid surface of the bondlayer, respectively. $d(x)$ is found out from the assumption of simple bending by taking integral of axial stresses in the multilayer and equating it to zero. The distance of the neutral axis from the mid-surface of the bondlayer over the bondlength is given by:

$$d\left( x \right)=\frac{\left( \frac{E_{xt}^{*}(x)}{1-v_{1}^{2}} \right)\left( h^{2}+ht \right)-\left( \frac{E_{xb}^{*}(x)}{1-v_{1}^{2}} \right)\left( h^{2}+ht \right)}{2h\left( \frac{E_{xt}^{*}(x)}{1-v_{1}^{2}} \right)+2h\left( \frac{E_{xb}^{*}(x)}{1-v_{1}^{2}} \right)+4t\left( \frac{E_{b}}{1-v_{b}^{2}} \right)}$$

The neutral axis is along the bondlayer midline in the overlap region for the baseline case, but becomes a function of 𝑥 with adherend tailoring. Bending stiffness variation over the overlap length of the joint for different cases is shown in Figure S10.


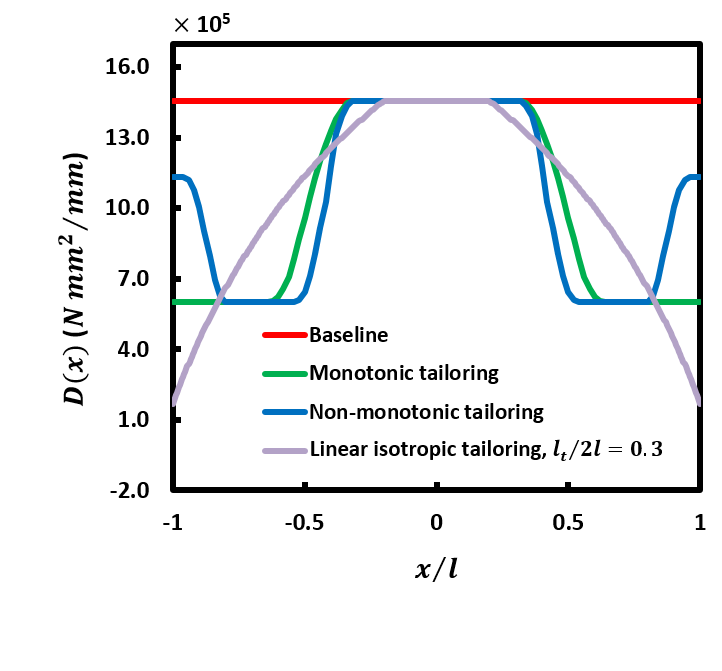


**Figure S10** Bending stiffness variation over the overlap length of SLJ multilayers with experimentally-realized adherend tailoring (monotonic and non-monotonic) compared to the linear tailoring with${l_{t}}/{2l}=0.3$ and ${E_{1}}/{E_{2}}=2000$ and the baseline case.

The bending moment per unit width in the multilayer is influenced by the compliance of the adherends, *e.g.,* increased compliance at the adherend ends allows rotation and reduces the bending moment. At the critical overlap ends, the bending moment per unit width in the adherends is evaluated by calculating the resultant moment due to axial stresses in the adherend at the overlap end from the finite element analysis:

$$M= \int_{y=\frac{t}{2}}^{y=h+t/2} (\sigma_{xx}-\sigma_{\infty}) \left( y-y_{0} \right) dy$$

where $\sigma_{xx}$and $\sigma_{\infty}$ are the axial stresses through the height of the top adherend at the overlap end ($y=-l$) and the applied stress, $y_{0}$ is the $y$ coordinate of the neutral axis where $\sigma_{xx}-\sigma_{\infty}=0$. The applied stress is subtracted from the axial stresses as it is not associated with the bending stresses. The bending moment at the overlap ends for $2l=50 mm$ and $2l\approx{2l}_{crit}$ ($\approx4m$) are calculated and tabulated in Table S3. Tailoring has a much more significant effect on bending moment for the longer overlap length and this is discussed in the main text.

**Table S3** Bending moment per unit width for $2l=50mm$ and $2l\approx{2l}_{crit}(\approx4m)$ for experimentally-realized adherend tailoring (monotonic and non-monotonic) compared to the linear tailoring with${l_{t}}/{2l}=0.3$ and ${E_{1}}/{E_{2}}=2000$ and the baseline case.

| **Adherend Design** | **Bending moment (**${\boldsymbol{N}\mathbf{*}\boldsymbol{m}}/\boldsymbol{m}$**)** | |
| --- | --- | --- |
|  | $\boldsymbol{2}\boldsymbol{l}\mathbf{=50}\boldsymbol{mm}$ | $\boldsymbol{2}\boldsymbol{l}\boldsymbol{\approx}{\boldsymbol{2}\boldsymbol{l}}_{\boldsymbol{crit}}$ |
| **Baseline** | 24.34 | 13.7 |
| **Monotonic tailoring** | 24.15 | 3.26 |
| **Non-monotonic tailoring** | 23.98 | 4.47 |
| **Linear isotropic tailoring,** ${\boldsymbol{l}_{\boldsymbol{t}}}/{\boldsymbol{2}\boldsymbol{l=0.3}}$ | 23.98 | 7.51 |

# **References**

1 ASTM D412-16, Standard Test Methods for Vulcanized Rubber and Thermoplastic Elastomers—Tension, ASTM International, West Conshohocken, PA, 2016, [www.astm.org](http://www.astm.org)

2 Cintron, R. & Saouma, V. Strain measurements with the digital image correlation system Vic-2D. *System* **106**, 2D (2008).

3 Ubaid, J., Kashfuddoja, M. & Ramji, M. Strength prediction and progressive failure analysis of carbon fiber reinforced polymer laminate with multiple interacting holes involving three dimensional finite element analysis and digital image correlation. *International Journal of Damage Mechanics* **23**, 609-635 (2014).

4 Koerber, H., Xavier, J. & Camanho, P. High strain rate characterisation of unidirectional carbon-epoxy IM7-8552 in transverse compression and in-plane shear using digital image correlation. *Mech. Mater.* **42**, 1004-1019 (2010).

5 Kendall, K. The adhesion and surface energy of elastic solids. *J. Phys. D: Appl. Phys.* **4**, 320, doi:10.1088/0022-3727/4/8/320 (1971).

6 Chung, J. Y. & Chaudhury, M. K. Soft and hard adhesion. *J. Adhes.* **81**, 1119-1145, doi:10.1080/00218460500310887 (2005).

7 Jackson, A., Vincent, J. & Turner, R. Comparison of nacre with other ceramic composites. *Journal of Materials Science* **25**, 3173-3178 (1990).

8 Brodt, M. & Lakes, R. S. Composite Materials Which Exhibit High Stiffness and High Viscoelastic Damping. *J. Compos. Mater.* **29**, 1823-1833, doi:10.1177/002199839502901402 (1995).

9 Jackson, A. P., Vincent, J. F. V. & Turner, R. M. The Mechanical Design of Nacre. *Proceedings of the Royal Society B: Biological Sciences* **234**, 415-440, doi:10.1098/rspb.1988.0056 (1988).

10 Volkersen, O. The rivet load distribution in lap-joints with members of constant thickness subjected to tension. *Luftfahrtforschung* **15**, 41-47 (1938).
